# The role of IT and English education in shaping cognitive and behavioral responses to green economic volatility

**DOI:** 10.3389/fpsyg.2026.1908334

**Published:** 2026-07-23

**Authors:** Huangjingcheng Yang

**Affiliations:** School of Liberal Arts, Renmin University of China, Beijing, China

**Keywords:** adaptive behavior, cognitive, English language, green economic volatility, IT

## Abstract

**Background:**

The transition to green economies requires individuals to interpret volatile sustainability-related information across digital platforms, international policy sources, and multilingual knowledge environments.

**Objective:**

This study examined how IT competency and English-language education are associated with cognitive and behavioral responses to green economic volatility.

**Methods:**

A sequential explanatory mixed-methods design was used. Quantitative survey data were collected from 420 academic and professional participants, followed by semi-structured interviews with 24 purposively selected participants. Regression and structural equation modeling (SEM) were used to test the hypothesized associations, while thematic analysis was used to explain and contextualize the quantitative findings.

**Results:**

IT competency was more strongly associated with cognitive response, including risk appraisal and information integration, whereas English proficiency was more strongly associated with adaptive behavioral response. The IT × English interaction was significant in both outcome models, indicating a synergistic association between digital and linguistic competencies.

**Conclusion:**

The findings support an interdisciplinary human-capital perspective in which digital and English-language competencies jointly strengthen individuals’ capacity to engage with sustainability-oriented economic uncertainty. The results inform curriculum design, green workforce development, and future research on education for sustainable economic participation.

## 1 Introduction

Economic transformation today is fundamentally shaped by the pursuit of environmental sustainability. The emergence of green economies encompassing low-carbon industries, markets for renewables, environmental governance systems, and sustainable finance tools has introduced a complex new layer to economic activity, demanding novel forms of understanding, adaptation, and decision-making by both individuals and institutions (Yang et al., 2025). However, this transformation has not followed a linear trajectory. A defining feature of the era is green economic volatility, seen in unpredictable fluctuations in the value of green assets, the policy environment, environmental regulation, and sustainable investment returns (Bolton et al., 2020). Successfully navigating this new landscape requires not just economic expertise but also a broad set of cognitive skills and educational backgrounds to help people comprehend, respond to, and adapt to volatile, sustainability-driven economies.

In this context, the competencies of information technology (IT) and English language education have come into prominence as two dimensions of human capital (Malik and Terzidis, 2025). Although these two domains appear to differ in their traditional academic and professional uses, their intersection in the context of the “Digital Green Economy” is an intriguing topic for interdisciplinary research. Cognitive literacy is essential, as financial markets, environmental data platforms, international climate policy discourses, and green investment ecosystems are increasingly becoming digital. At the same time, English language skills are essential in the scientific and academic world, as the primary language for international environmental agreements, digital information, and financial reporting (Dote Pardo, 2025). Further, English language skills are playing an increasingly important role as a gateway to accessing, processing, and applying green economic knowledge, while also serving as the dominant language of the global scientific and academic world, financial reporting, international environmental agreements, and digital information. Combined, these two learning skills are suspected to influence the perceptions of risk greatly, the building of understanding, and ultimately the behavioral response of individuals to situations of green economic uncertainty (van Laar et al., 2017).

Recent research largely focuses separately on green finance and human capital development, but very few studies have examined how IT and English interact to shape cognitive and behavioral responses within the volatile green economic context. While the literature considers financial literacy’s effect on investment choices, the economic value of digital skills, and how language education facilitates global market access, the connection between educational competencies, cognitive processing of economic information, and behavioral adaptation to market greening remains underexplored (World Bank Group, 2016). This study addresses this gap by examining IT education and English language teaching not just as instrumental skills, but as cognitive frameworks that influence how individuals interpret and engage with the complex realities of the green economic transition. More specifically, three distinct literatures bear on this question: research on financial and digital literacy in relation to economic decision-making (Lusardi and Mitchell, 2014); work on the economic returns to digital skills in information-intensive settings (Leicht and Byun, 2021); and scholarship on language proficiency as a means of accessing globally produced scientific, regulatory, and financial knowledge (Ajzen, 1991). These literatures have developed largely in isolation, and comparatively little work has examined how IT competency and English proficiency jointly shape cognitive and behavioral responses to specifically green economic volatility.

This research integrates human capital theory, cognitive adaptation, behavioral economics, technology-mediated learning, and second-language acquisition to build a coherent theoretical foundation (Schultz, 1961). Human capital theory positions IT competency and English proficiency as educational resources that can improve access to, interpretation of, and action on complex green-market information. Research on computer-assisted language learning and multimedia learning further suggests that digital interfaces, visual information, and language comprehension shape how learners process technical knowledge and apply it in real-world contexts (Mayer, 2017). Within this framework, IT competency is expected to support data search, filtering, visualization, and analytical processing, while English proficiency is expected to expand access to global scientific, regulatory, and financial sources. Green economic volatility provides the empirical context because it combines financial uncertainty with policy, technological, and interpretive uncertainty. The study therefore tests whether these competencies are associated with cognitive response, behavioral response, their interaction, and the mediating role of perceived green economic volatility (Slovic, 1987).

The green economy poses a special cognitive challenge. Green markets differ from traditional ones. They are shaped by environmental science, policy, technology, and social values (Besonia, 2024). This removes a historical record and a clear interpretive framework. Green economic volatility is not only financial; it is also informational and interpretive. To succeed, one must integrate technical, scientific, regulatory, and financial information in English within digital spaces. Digital platforms, data visualization, and online financial tools give those with IT skills a chance to find, filter, and analyze information (Arner et al., 2016). For those with strong English skills, the knowledge base is broader: climate reports, green bond prospectuses, environmental impact assessments, and sustainability indices support informed and context-specific responses to sustainability issues.

The behavioral aspect of this study is also significant. No cognitive reaction to economic volatility is isolated; it manifests in behavioral outcomes such as investment, career decisions, organizational strategies, policy advocacy, and consumption. Knowing how educational competency translates from cognitive processing to behavioral outcomes is vital for academic study and practical policy in education (Gonzalez and Ramos, 2026). The result has broad implications for curriculum design, workforce development policies, and investment strategies in green transition economies, as prior work suggests that digital competence and sustainability-oriented education may support individuals’ ability to adapt to green economic volatility (Bolton et al., 2020). The preparation of citizens for sustainable economic participation cannot be limited to environmental awareness and technical-vocational education, but also involves developing a general cognitive framework that facilitates citizens’ functioning in a complex, dynamic, and information-intensive economic environment (OECD, 2019).

This study addresses three related goals. First, it examines the association between IT competency and cognitive responses to green economic volatility, including risk perception and information processing. Second, it assesses whether English-language proficiency is associated with access to green economic knowledge and adaptive behavioral responses to market uncertainty. Third, it evaluates whether the IT × English interaction is associated with stronger cognitive flexibility and behavioral adaptability in green economic contexts. The sequential explanatory mixed-methods design was selected to combine model-based evidence with qualitative explanation of the mechanisms underlying these associations.

## 2 Research methodology

### 2.1 Overview of research design

This study used a sequential explanatory mixed-methods design in which quantitative data were collected and analyzed first, followed by qualitative interviews designed to explain and contextualize the statistical results. This design was appropriate because the research questions required both the statistical capacity to estimate associations among IT competency, English proficiency, green economic volatility perception, and adaptive responses, and the interpretive capacity to understand how participants experienced these relationships in practice. The pragmatic paradigm guided the integration of these methods, allowing the study to examine green economic volatility as both an objective market condition and a subjectively interpreted educational and behavioral challenge.

### 2.2 Research philosophy and paradigm

This study is based on the epistemological paradigm of pragmatism, which emphasizes practical outcomes and the study’s applicability in reality rather than any particular methodological tradition. The mixed-methods approach is embraced by pragmatism, which acknowledges that quantitative data and qualitative responses add value to the process of constructing knowledge. Ontologically, this study recognizes that green economic volatility is an objective market phenomenon and a subjectively experienced condition, the perception and interpretation of which are influenced by individual cognitive frameworks shaped by education, experience, and cultural context. It is a dual ontological positioning that allows for both structured quantitative instruments and open-ended qualitative inquiries to be used within the same research framework.

### 2.3 Research hypotheses

Based on the theoretical framework and literature review, five hypotheses were specified. H1: Higher IT competency is positively associated with stronger cognitive responses to green economic volatility, including risk-perception accuracy and information-integration capacity. H2: Higher English proficiency is positively associated with greater access to green economic knowledge and more adaptive behavioral responses to green market uncertainty. H3: The IT × English interaction is positively associated with cognitive flexibility and behavioral adaptability in green economic contexts. H4 (control-variable hypothesis): Age, gender, and educational level explain additional variance in cognitive and behavioral responses after adjustment for IT competency, English proficiency, green economic volatility perception, and the IT × English interaction. H5: Perception of green economic volatility partially mediates the associations between IT competency, English proficiency, and cognitive and behavioral responses. This formulation aligns the theoretical propositions with the statistical model by treating demographic characteristics as control variables used to adjust for demographic heterogeneity.

### 2.4 Study population and sampling strategy

#### 2.4.1 Target population

The scope of this study is people actively involved in an educational or professional setting where green economic knowledge is relevant. This encompasses students at university studying economics, environmental science, business, or technical subjects, as well as those in jobs directly related to sustainability, green finance, environmental policy, and information technology. It is assumed that residents in these domains are most likely to regularly access green economic information and that they have differing levels of IT and English language proficiency, both of which are relevant to the research variables.

#### 2.4.2 Sample size and justification

In the quantitative phase of the study, 420 participants were recruited. The *a priori* sample size was determined using the software G*Power, which revealed that a minimum sample size of 385 participants was needed to achieve a statistical power of 0.80 at a significance level of *α* = 0.05 and with a medium effect size (*f*^2^ = 0.15) for the multiple regression analysis with up to 8 predictor variables. The sample size *n* = 420 was thus chosen as a buffer above the minimum required, and would also allow for some attrition due to incomplete responses or participant drop-out. For the qualitative phase, 24 respondents from the quantitative phase were selected to achieve maximum variation in IT competency, English language proficiency, professional background, and demographic characteristics. This sample size aligns with those typical of qualitative research, with theoretical saturation (when no new themes emerge from the data when the analysis is compared to the collected data) generally occurring in one’s interview-based study with 20–30 participants.

#### 2.4.3 Sampling technique

A stratified sampling strategy was used in the quantitative phase to ensure that the sample reflected the main academic and professional groups relevant to the study topic. Recruitment was conducted in Beijing, China, through the School of Liberal Arts, Renmin University of China, and cooperating academic and professional networks related to education, finance, technology, sustainability, and environmental governance. Eligible participants included university students and professionals who had prior exposure to green economic topics, digital information systems, English-language academic or professional materials, or sustainability-related decision-making contexts.

The sampling frame was organized according to five key strata: gender, educational level, professional sector, prior exposure to green economic topics, and level of engagement with sustainability-related academic or professional activities. Participants were then selected proportionally from these strata to reduce sampling bias and to improve representation across academic and professional backgrounds. The final quantitative sample consisted of 420 participants.

For the qualitative phase, purposive sampling was applied after the preliminary analysis of the quantitative data. Twenty-four participants were selected to represent variation in IT competency, English proficiency, professional background, demographic characteristics, and response patterns. This maximum-variation approach allowed the qualitative interviews to capture contrasting experiences, including participants with high and low levels of IT competency and English proficiency, as well as participants showing stronger or weaker cognitive and behavioral responses to green economic volatility. This sampling approach was appropriate for the sequential explanatory mixed-methods design because the qualitative phase was used to explain and contextualize the quantitative findings.

### 2.5 Data collection instruments

#### 2.5.1 Quantitative instrument: structured survey questionnaire

The quantitative instrument was a structured, self-administered questionnaire developed for the present study. It contained six sections covering demographic and background information, IT competency, English proficiency, green economic volatility perception, cognitive response, and behavioral response. Demographic items captured age, gender, nationality, highest educational qualification, field of study or professional specialization, professional experience, and prior engagement with green economic topics. These variables were retained as statistical controls in the regression and SEM analyses. Section B measured IT competency using items adapted primarily from the European Commission Digital Competence Framework (DigComp 2.2).

The scale consisted of 20 items spanning the following five sub-dimensions: information and data literacy, communication and collaboration using digital tools, digital content creation, cybersecurity awareness, and problem-solving with technology. Items were scored on a scale of 1 to 5, with 1 being strongly disagree and 5 being strongly agree. Sample items are: “I can search, filter, and assess green economic information on the Internet with confidence,” and “I can analyze environmental market-trend data visualizations with confidence.” English language proficiency was measured using a composite scale that included a short objective language comprehension task and a self-assessed language proficiency scale based partly on CEFR descriptors. The self-assessment component consisted of 15 items from the Common European Framework of Reference for Languages (CEFR) self-assessment grid, divided into four domains: reading, listening, speaking, and writing. The goal-oriented component included three short passages from real-world green economic documents (including a part from a green bond framework document, an excerpt from an IPCC summary report, and a paragraph from an international sustainability index) and included comprehension and inference questions. Both parts of the test were aggregated to form a composite measure of English proficiency. Section D: Green Economic Volatility Perception Scale. This scale aims to measure cognitive awareness and perception of green economic volatility, its causes, manifestations, and implications. This scale was developed based on a conceptual framework on environmental risk perception and adapted to green financial markets.

The items covered were related to how unpredictable people perceived green assets to be, how aware they were of policy-driven market changes, and how complex they found green investment environments. A five-point Likert scale was used to rate all items. In response to green economic volatility, cognitive responses were categorized into three dimensions: risk perception accuracy, information integration capacity, and cognitive flexibility. Based on scales employed in cognitive economics studies, and adapted to the green economic framework, a 22-item scale was created. Sample questions: When I run into conflicting information about the performance of the green market, I can critically assess the sources and make a balanced judgment; when I face changes in environmental regulations that are complex, I feel cognitively prepared to interpret the changes and understand the economic implications. The four domains for behavioral responses were green investment behavior, information-seeking behavior in response to market uncertainty, advocacy behavior to support the implementation of sustainable economic policy, and occupational or academic adaptation behavior. The 20-item scale was based on behavioral economics theory and the Theory of Planned Behavior, which includes intention-related and action-related behavioral indicators. Items were rated using a combination of a Likert scale and item frequency.

#### 2.5.2 Qualitative instrument: semi-structured interview protocol

Qualitative data were collected through semi-structured individual interviews with 24 purposively selected participants. The interview protocol consisted of 12 open-ended questions, which were grouped under three thematic areas, namely: (1) how IT skills help in adapting to green economic uncertainty; (2) how English language proficiency helps to access green economic knowledge; and (3) what kinds of cognitive and behavioral adaptations are being made in the face of green economic volatility? The questions were designed to prompt elaboration and to develop new themes. Interviews were held via secure video conferencing for 45–75 min. The interviews were all audio-recorded with participants’ informed consent, then transcribed verbatim for qualitative analysis.

### 2.6 Pilot testing and instrument validation

The survey questionnaire was rigorously piloted with 35 individuals from the target population, but not included in the main sample. The clarity of item wording, the appropriateness of response formats, and the estimated completion time for each scale were assessed through pilot testing, as were the preliminary psychometric properties of each scale. Following pilot testing, seven items were modified for clarity, two were deleted as redundant, and Section C was reorganized to reduce cognitive fatigue. Cronbach’s alpha coefficient was used to determine the reliability of quantitative scales. The alpha values of all the final scales were above 0.70, the acceptable level, ranging from *α* = 0.76 for the English proficiency self-assessment component to *α* = 0.89 for the cognitive response scale. Content validity was assessed through expert item analysis (five academics with expertise in green economics, educational psychology, digital literacy, and applied linguistics) on the relevance, representativeness, and clarity of the items. Confirmatory factor analysis (CFA) was performed to test construct validity, and the hypothesized factor structure for each scale was confirmed. The pilot sample (*n* = 35) was used only for item refinement and reliability screening; the CFA was estimated on the full study sample (*N* = 420). The measurement model showed acceptable fit (*χ*^2^/df = 2.06; CFI = 0.958; TLI = 0.947; RMSEA = 0.050; SRMR = 0.047). Standardized factor loadings ranged from 0.61 to 0.89, all significant at *p* < 0.001. Convergent validity was supported, with composite reliability between 0.78 and 0.91 and average variance extracted at or above 50 for all constructs; discriminant validity was confirmed using the HTMT ratio, with all HTMT values below 85.

### 2.7 Data collection procedure

Data were collected quantitatively over 8 weeks via an online survey platform. The survey link was distributed to eligible respondents via official email communications, leveraging institutional cooperation established with three universities and two professional organizations. All participation was voluntary, and informed consent was obtained electronically prior to the survey. To reduce response bias, participants were informed that their responses would be anonymous and that the research was for academic purposes only. Qualitative data analysis was conducted in weeks 9–14 of data collection after quantitative data analysis. Participants for the interviews were contacted individually by e-mail and provided with an information sheet outlining the purpose, structure, and confidentiality of the interview. To avoid language as a barrier to meaningful participation in the interviews, all interviews were conducted in English, with bilingual support provided upon request.

### 2.8 Data analysis strategy

#### 2.8.1 Quantitative analysis

The quantitative data were analyzed using IBM SPSS Statistics Version 28 and AMOS Version 26. Descriptive statistics were calculated for all study variables, and normality was assessed using the Kolmogorov–Smirnov test. Bivariate relationships among the major variables were examined using Pearson correlation analysis. Hierarchical multiple regression was then used to estimate the associations of IT competency, English proficiency, green economic volatility perception, and the IT × English interaction with cognitive and behavioral responses while adjusting for age, gender, and educational level. These demographic variables were entered as covariates because the revised theoretical model treats them as background characteristics that may account for additional variance, rather than as explanatory mechanisms. This analytical decision ensures consistency between the hypotheses and the statistical implementation, as recommended for transparent SEM reporting (Mohammadian, 2020; Mayer et al., 2005). Structural equation modeling was used to evaluate the full theoretical model, including direct paths, the IT × English interaction, the mediating role of green economic volatility perception, and demographic covariate paths.

#### 2.8.2 Qualitative analysis

The qualitative interview data were analyzed using thematic analysis in six phases: data familiarization, initial coding, theme development, theme review, theme definition and naming, and final reporting (Zhang and Zou, 2022). Transcripts were managed and coded in NVivo Version 14. Coding began inductively to allow participant-generated meanings to emerge and was then interpreted deductively in relation to the study’s theoretical framework. Thirty percent of transcripts were coded by a second independent coder to assess inter-rater reliability, yielding Cohen’s kappa = 0.82, indicating strong coding agreement.

### 2.9 Ethical considerations

This study involved human participants and was conducted in accordance with the Declaration of Helsinki and accepted ethical principles for social science research (World Medical Association, 2013). The human-participant component comprised 420 survey participants and 24 semi-structured interview participants. Ethical approval was obtained from the Research Ethics Committee of Renmin University of China before data collection. The study was approved under approval letter number SSU-REC-2026-028 on March 17, 2026. The approved documents included the research plan, informed consent form, questionnaire, participant information sheet, and data confidentiality statement.

All participants received information about the study purpose, procedures, voluntary participation, confidentiality protections, and the right to withdraw at any time without penalty. Electronic informed consent was obtained before survey participation, and separate informed consent was obtained before the qualitative interviews and audio recording. No personally identifiable information is reported in the manuscript. Survey responses were anonymized using identification codes, and interview transcripts were de-identified before analysis. Audio recordings and research data were stored on password-protected and encrypted institutional devices accessible only to the research team. The manuscript and submission materials should therefore consistently indicate that the study involved human participants.

### 2.10 Limitations of the methodology

The mixed-methods design enhances the rigor and comprehensiveness of this study, but some methodological issues must be noted. The first is the use of self-reported measures of IT and English-language competency, which could lead to social desirability bias, as participants might overestimate their competency. While the objective English comprehension task somewhat reduces this drawback, future studies could well benefit from the use of performance-based IT assessments. Second, because the quantitative study used a cross-sectional design, causal inferences cannot be drawn; associations between variables cannot be interpreted as causal. Thirdly, although the sample is broad, it is also drawn from academic and professionally engaged populations, which may limit the generalizability of the findings to other, and/or less academic, demographic groups. Fourth, because the predictor and outcome variables were measured with the same self-report instrument, part of the observed strength of the associations may reflect shared method variance; for this reason common method bias was assessed using Harman’s single-factor test and multicollinearity was examined through variance inflation factors, and the reported effect sizes should be interpreted with this measurement context in mind.

## 3 Results and discussion

The empirical results are presented in a sequenced manner consistent with the explanatory mixed-methods design: descriptive statistics and reliability coefficients, bivariate correlations, hierarchical regression, SEM, and qualitative thematic analysis (Leguina, 2015). Quantitative results are reported first to test the hypotheses, and qualitative findings are then used to explain the observed patterns. This structure improves transparency by separating statistical estimation from interpretive explanation while preserving mixed-methods integration.

### 3.1 Descriptive statistics and reliability analysis

Table 1 shows descriptive statistics for all primary study variables: mean, standard deviation, minimum and maximum values, and Cronbach’s alpha reliability coefficients. The internal consistency of all scales was satisfactory to excellent, ranging from *α* = 0.76 for the English proficiency self-assessment component to α = 0.89 for the overall cognitive response scale (Thorndike, 1995). The average overall IT competency score was *M* = 3.61 (SD = 0.73), suggesting that participants had moderate digital literacy. The five sub-dimensions had different sub-scale means, with Problem-Solving via Technology (*M* = 3.76, SD = 0.70) having the highest and Digital Content Creation (*M* = 3.44, SD = 0.79) having the lowest. Overall, English language proficiency achieved a composite index mean score of *M* = 3.48 (SD = 0.81), where the reading comprehension subscale had a higher mean score (*M* = 3.62, SD = 0.79) than the objective comprehension subscale (*M* = 3.31, SD = 0.88), indicating a tendency toward self-assessment inflation, in line with the social desirability bias noted in the methodology (Becker, 1993).

**Table 1:** Descriptive statistics and internal reliability coefficients for study variables (*N* = 420).

Variable	*N*	*M*	SD	Min	Max	*α*
IT competency (total)	420	3.61	0.73	1.60	5.00	0.87
Information and data literacy	420	3.72	0.68	1.80	5.00	0.83
Communication and collaboration	420	3.58	0.71	1.60	5.00	0.80
Digital content creation	420	3.44	0.79	1.40	5.00	0.81
Cybersecurity awareness	420	3.55	0.76	1.60	5.00	0.79
Problem-solving via technology	420	3.76	0.70	1.80	5.00	0.84
English proficiency (composite index)	420	3.48	0.81	1.33	5.00	0.82
Reading comprehension (CEFR)	420	3.62	0.79	1.50	5.00	0.80
Objective comprehension score	420	3.31	0.88	1.00	5.00	0.76
GEV perception scale	420	3.39	0.77	1.44	5.00	0.84
Cognitive response scale (Total)	420	3.55	0.74	1.45	5.00	0.89
Risk perception accuracy	420	3.48	0.76	1.33	5.00	0.85
Information integration capacity	420	3.61	0.71	1.60	5.00	0.86
Cognitive flexibility	420	3.57	0.75	1.40	5.00	0.87
Behavioral response scale (total)	420	3.44	0.79	1.35	5.00	0.88
Green investment behavior	420	3.37	0.83	1.25	5.00	0.85
Information-seeking behavior	420	3.53	0.77	1.40	5.00	0.83
Policy advocacy behavior	420	3.41	0.82	1.20	5.00	0.82
Occupational adaptation behavior	420	3.45	0.78	1.30	5.00	0.84

M, mean; SD, standard deviation; α, Cronbach’s alpha; GEV, green economic volatility; CEFR, common European framework of reference. All scales were measured on a 1–5 Likert scale. Sub-scale items are indented.

**Figure 1:**
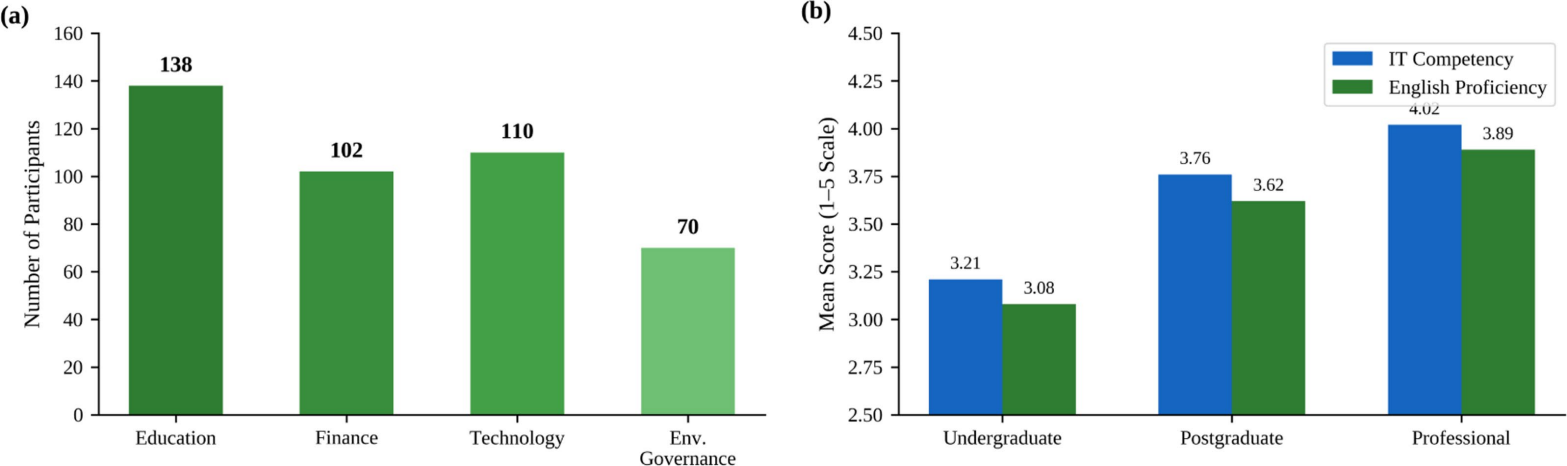
Sample distribution by professional sector **(a)** and mean IT competency and English proficiency scores by educational level **(b)**. Error bars represent ±1 SD. Differences across educational levels were statistically significant for both variables (*p* < 0.001).

Participants’ awareness of green market instability was moderate to moderately high, with a Green Economic Volatility (GEV) Perception score of *M* = 3.39 (SD = 0.77). Cognitive response scores were *M* = 3.55 (SD = 0.74) on the total scale, and cognitive flexibility was slightly higher (*M* = 3.57) than perception of risk accuracy (*M* = 3.48) (Prokopenko et al., 2025). Results show that behavioral response scores have been averaged at *M* = 3.44 (SD = 0.79), with information-seeking behavior scoring highest on the sub-scale (*M* = 3.53) and green investment behavior scoring lowest (*M* = 3.37), indicating that the participants are more motivated to engage in information rather than direct financial action when dealing with green market uncertainty (Prokopenko et al., 2025) (Figure 1).

### 3.2 Bivariate correlation analysis

**Figure 2:**
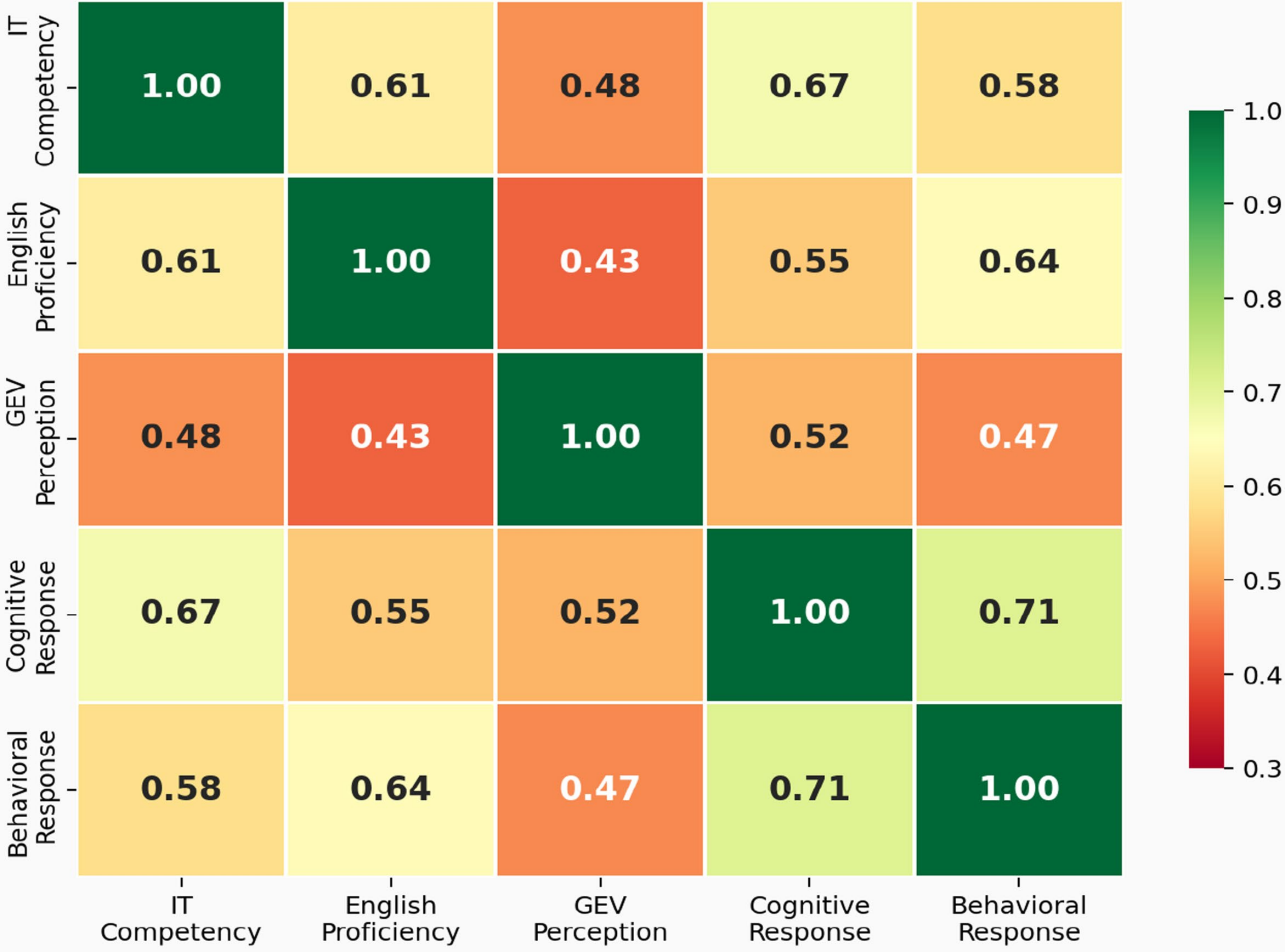
Pearson correlation matrix for primary study variables (*N* = 420). Cell values represent Pearson r coefficients. All correlations are statistically significant at *p* < 0.001. IT, information technology competency; EP, English proficiency; GEV, green economic volatility perception; CR, cognitive response; BR, behavioral response.

The bivariate relationships among the five major study variables were examined using Pearson correlation coefficients. The results of all correlations were significant at the *p* < 0.001 level as shown in Figure 2. For cognitive responses, there was a strong positive correlation with IT competency (*r* = 0.67, *p* < 0.001), indicating a strong association between cognitive responses to green economic volatility and IT competency scores (Yiming et al., 2024). The level of English was most strongly correlated with behavioral response (*r* = 0.64, *p* < 0.001), as expected, because knowledge about global green markets is more likely to translate into behavioral response than into cognitive processing capacity alone. The correlation between IT competency and English proficiency (*r* = 0.61, *p* < 0.001) was significant, indicating conceptual and functional overlap between the two constructs, yet they remained empirically distinguishable (Loewenstein et al., 2001). GEV perception was moderately related to both cognitive (*r* = 0.52, *p* < 0.001) and behavioral responses (*r* = 0.47, *p* < 0.001) and thus plays an important, but not overwhelming, role in both response types. The strongest relationship in the matrix was between cognitive and behavioral responses (*r* = 0.71, *p* < 0.001), highlighting their relation in the context of green economic engagement and reinforcing the theoretical model’s distinction between them (Zhylin et al., 2025).

### 3.3 Multiple regression analysis

#### 3.3.1 Predicting cognitive response (H1 and H3)

A hierarchical multiple regression analysis was used to test the contributions of IT competency and English proficiency, GEV perception, and the IT–English interaction to cognitive response scores, while controlling for age, gender, and educational level (Kline, 2023). The full model accounted for a significant and large amount of variance in cognitive responses: *R*^2^ = 0.624, *F*(7, 412) = 97.68, *p* < 0.001, indicating that the predictor set as a whole accounted for 62.4% of the variance. IT competency was the strongest individual predictor of cognitive response (*β* = 0.41, *t* = 8.67, *p* < 0.001, sr^2^ = 0.112), which supported Hypothesis 1 (H1) (Stern, 2000). The results indicate that participants with higher digital literacy showed greater risk perception accuracy, stronger information integration capacity, and greater cognitive flexibility regarding green economic volatility.

**Figure 3:**
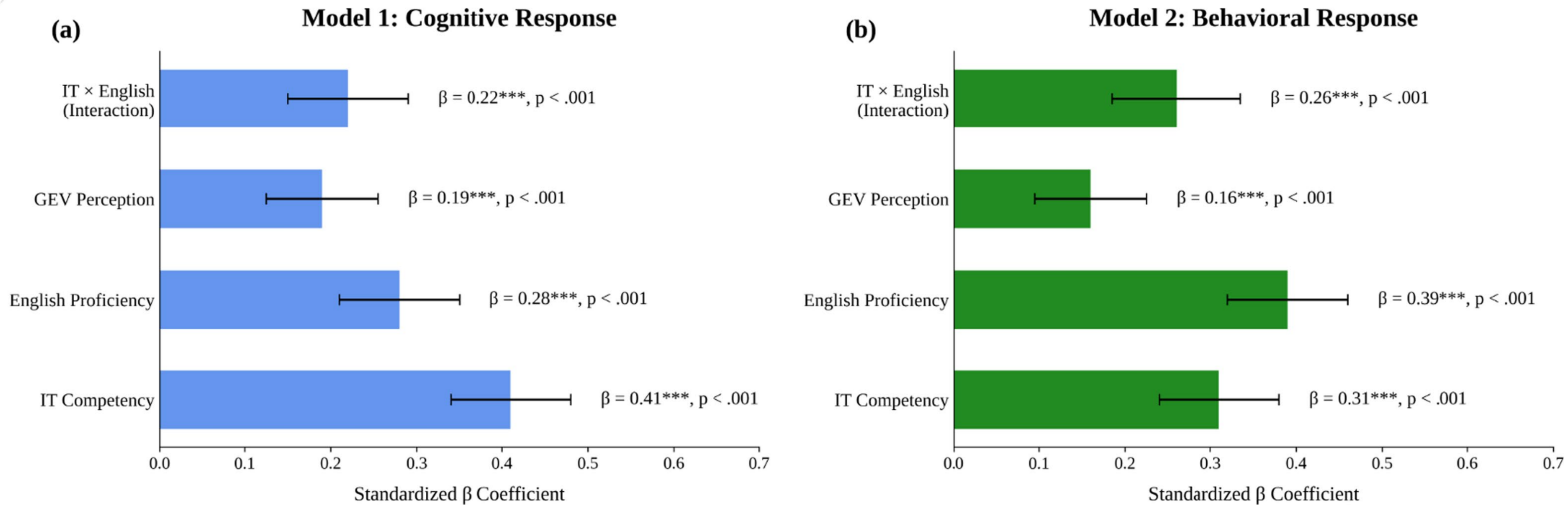
Standardized regression coefficients (β) with 95% confidence intervals for Model 1 (Cognitive Response; **a**) and Model 2 (Behavioral Response; **b**). Horizontal error bars represent 95% CIs. All predictors labeled *** are significant at *p* < 0.001.

English proficiency was also significantly associated with cognitive response (*β* = 0.28, *t* = 6.12, *p* < 0.001, sr^2^ = 0.067), although its contribution was lower than that of IT competency for this outcome. Green economic volatility perception was positively associated with cognitive response (*β* = 0.19, *t* = 4.28, *p* < 0.001, sr^2^ = 0.033), indicating that stronger awareness of green-market instability was linked to better cognitive appraisal (Wang et al., 2020). The IT × English interaction was significant (*β* = 0.22, *t* = 4.79, *p* < 0.001, sr^2^ = 0.041), supporting H3. Educational level, entered as a demographic control variable, was significant in predicting cognitive outcomes (*β* = 0.08, *t* = 2.14, *p* = 0.033), whereas age (*β* = 0.07, *p* = 0.057) and gender (*β* = 0.04, *p* = 0.278) were not significant. These results support the control-variable formulation of H4 by showing a modest independent contribution of educational level after adjustment for the main predictors (Figure 3).

#### 3.3.2 Predicting behavioral response (H2 and H3)

The second regression model predicting behavioral response yielded *R*^2^ = 0.591, *F*(7, 412) = 85.05, *p* < 0.001, with the predictor set accounting for 59.1% of the variance in behavioral response. When comparing predictor dominance with the cognitive model, English proficiency was the most significant predictor of behavioral response (*β* = 0.39, *t* = 8.16, *p* < 0.001, sr^2^ = 0.120), exceeding the contribution of IT competency (*β* = 0.31, *t* = 6.47, *p* < 0.001, sr^2^ = 0.076).

This pattern is theoretically coherent and is consistent with Hypothesis 2 (H2), where English language access to global green economic intelligence, such as international green finance documents, policies, and reports, translates most directly into behavioral adaptation, such as green investment engagement, information seeking, policy advocacy, and occupational adaptation (Schultz, 1961). The hypothesized compounding effect in H3 was further supported by a significant interaction between IT and English in the behavioral model (*β* = 0.26, *t* = 5.59, *p* < 0.001, sr^2^ = 0.056). GEV perception was positively associated, but weakly (*β* = 0.16, *t* = 3.53, *p* < 0.001, sr^2^ = 0.022), with behavioral outcomes (Tran and Vo Thai, 2025). Gender approached significance (*β* = 0.07, *p* = 0.069), and again, educational level was a significant covariate (*β* = 0.09, *p* = 0.018). Overall, these findings validate differential effects of IT and English skills by outcome type and a constant synergistic relationship between the two skill types across both outcome domains (Table 2).

**Table 2:** Hierarchical regression models predicting cognitive and behavioral responses (*N* = 420).

Predictor	*B*	SE	*β*	*t*	*p*	95% CI lower	95% CI upper	sr^2^
Model 1: cognitive response — *R*^2^ = 0.624, Δ*R*^2^ = 0.618, *F*(7, 412) = 97.68, *p* < 0.001								
IT competency	0.43	0.05	0.41	8.67	<0.001	0.34	0.53	0.112
English proficiency	0.29	0.05	0.28	6.12	<0.001	0.20	0.38	0.067
GEV perception	0.20	0.05	0.19	4.28	<0.001	0.11	0.29	0.033
IT × English interaction	0.23	0.05	0.22	4.79	<0.001	0.14	0.33	0.041
Age (covariate)	0.06	0.03	0.07	1.91	0.057	−0.00	0.12	0.006
Gender (covariate)	0.04	0.04	0.04	1.09	0.278	−0.04	0.12	0.002
Educational level (cov.)	0.08	0.04	0.08	2.14	0.033	0.01	0.16	0.008
Model 2: behavioral response — *R*^2^ = 0.591, Δ*R*^2^ = 0.584, *F*(7, 412) = 85.05, *p* < 0.001								
IT competency	0.32	0.05	0.31	6.47	<0.001	0.22	0.42	0.076
English proficiency	0.41	0.05	0.39	8.16	<0.001	0.31	0.51	0.120
GEV perception	0.17	0.05	0.16	3.53	<0.001	0.07	0.27	0.022
IT × English interaction	0.27	0.05	0.26	5.59	<0.001	0.17	0.37	0.056
Age (covariate)	0.05	0.03	0.06	1.71	0.088	−0.01	0.11	0.004
Gender (covariate)	0.07	0.04	0.07	1.82	0.069	−0.01	0.14	0.006
Educational level (cov.)	0.09	0.04	0.09	2.38	0.018	0.02	0.17	0.010

B, unstandardized coefficient; SE, standard error; β, standardized coefficient; sr^2^, squared semi-partial correlation; CI, confidence interval. Age, gender, and educational level were included as demographic covariates in both models. This table presents covariate-adjusted associations and does not test conditional demographic effects. ***p < 0.001, **p < 0.01, *p < 0.05.

### 3.4 Structural equation modeling (SEM) results

A structural equation model (SEM) was estimated using maximum likelihood estimation in IBM SPSS AMOS 26 to examine the hypothesized relationships among the constructs simultaneously. The model included IT competency and English proficiency as exogenous latent predictors, green economic volatility perception (GEV perception) as a mediating construct, the IT × English interaction as a product term, and cognitive response and behavioral response as correlated endogenous outcomes. Age, gender, and educational level were included as demographic covariates on the outcome paths, consistent with the hierarchical regression models.

**Figure 4:**
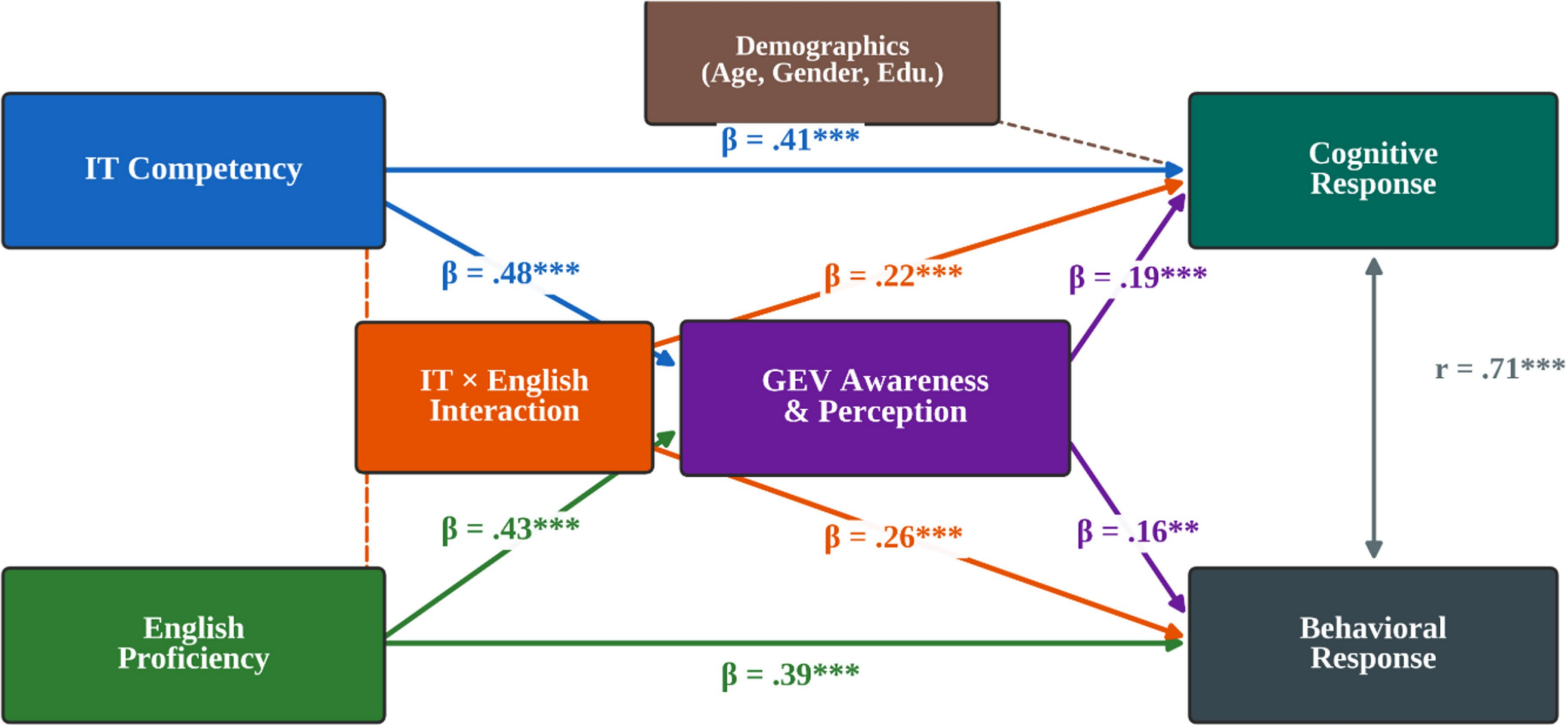
Structural equation model path diagram with standardized path coefficients. All paths labeled *** are significant at *p* < 0.001. IT, IT competency; EP, English proficiency; GEV, green economic volatility perception; Dem, demographic covariates; CR, cognitive response; BR, behavioral response. The figure label ‘GEV awareness and perception’ corresponds to the GEV perception construct used throughout the manuscript. ** indicates the statistical significance level of the corresponding path coefficient.

The model showed good overall fit: *χ*^2^/df = 2.14 (*χ*^2^ = 312.44, df = 146), CFI = 0.963, TLI = 0.951, RMSEA = 0.052 (90% CI: 0.041–0.063), and SRMR = 0.048. These indices met widely used SEM fit criteria (Zhang and Zou, 2022; World Medical Association, 2013). The standardized structural paths were consistent with the regression results. IT competency was more strongly associated with cognitive response (*β* = 0.41, *p* < 0.001) than with behavioral response (*β* = 0.31, *p* < 0.001), whereas English proficiency was more strongly associated with behavioral response (*β* = 0.39, *p* < 0.001) than with cognitive response (*β* = 0.28, *p* < 0.001). The indirect effects through GEV perception, interpreted according to mediation-analysis guidance, were significant for both outcomes (indirect *β* = 0.09 for cognitive response; indirect *β* = 0.07 for behavioral response). Because the direct paths remained significant, these findings indicate partial mediation. The residual correlation between cognitive and behavioral responses was significant (*r* = 0.71, *p* < 0.001), supporting their treatment as related but distinct outcomes in the model (Figure 4).

### 3.5 Qualitative findings: thematic analysis

**Figure 5:**
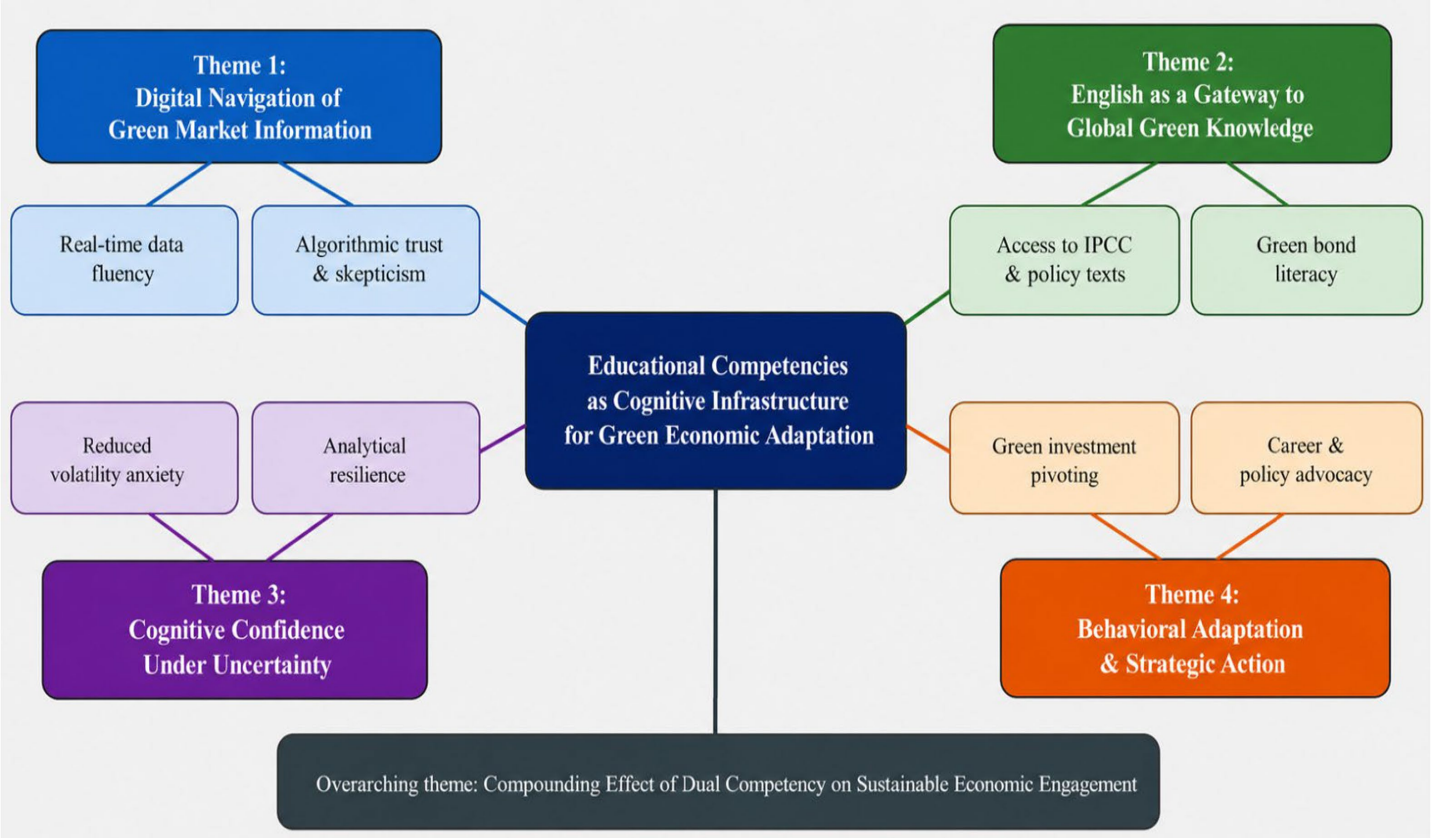
Thematic map of qualitative findings derived from semi-structured interview analysis (*N* = 24). Colored boxes represent primary themes; lighter sub-boxes represent sub-themes within each theme; connecting arrows indicate thematic relationships. The dark base panel represents the overarching theme unifying all primary themes.

The 24 semi-structured interviews were analyzed for themes, yielding four main themes and one overall theme that enhanced and explained the quantitative results. These themes are summarized in Figure 5 and will be discussed further below. An independent coder checked interrater reliability on 30% of the transcripts, yielding a Cohen’s Kappa of *κ* = 0.82, which was deemed good.

#### 3.5.1 Theme 1: digital navigation of green market information

The first theme reflects participants’ experiences using IT to find, screen, and critically assess green economic information in the context of such fluid digital environments. Investors with more experience in IT said they used specialized platforms to monitor market signals and policy changes, such as Bloomberg Green, MSCI ESG indices, and real-time environmental bond dashboards. A related sub-theme, referred to here as real-time data fluency, concerned the participants’ ability to understand financial instruments and real-time data visualizations (Hu and Bentler, 1999). Among technically sophisticated participants, a contrasting sub-theme emerged: algorithmic trust and skepticism, revealing a nuanced understanding of the challenges posed by automated data aggregation tools and the need to critically assess data sources. The low-IT partakers, on the other hand, reported suffering from “information paralysis” amidst market turbulence when confronted with unfamiliar digital financial interfaces.

#### 3.5.2 Theme 2: English as a gateway to global green knowledge

The second theme highlights the role of English in bridging primary and authoritative sources of green economic knowledge. For those with higher levels of English proficiency, the ability to directly interact with the IPCC’s assessment reports, the green bond frameworks of international financial institutions, and the sustainability disclosure reports of multinational corporations was consistently mentioned (Hayes, 2017). The sub-theme of access to IPCC and policy texts indicated that individuals who participated in English were able to access technical, scientific, and regulatory texts that were not available in translation, which allowed them to respond to them in a more informed and globally contextualized manner. The sub-theme of green bond literacy arose from anecdotes from participants who had had to wade through lengthy prospectus documents and environmental covenants to make investment decisions, suggesting that those with lower levels of understanding were unable to access such information. Several people explicitly stated the compounding nature of IT and English, saying that online platforms were most beneficial when they could read and interpret the English-language content they surfaced.

#### 3.5.3 Theme 3: cognitive confidence under uncertainty

The third theme focuses on the subjective experiences of cognitive confidence amid green economic volatility. Strong IT and English communicators identified a qualitative difference in how participants responded to market uncertainty: participants engaged in the market with a more informed analysis and measured responses rather than avoidance or anxiety (Fetters et al., 2013). Two opposing cognitive postures emerged from the sub-theme analysis: reduced volatility anxiety (highly competent participants’ ability to contextualize market fluctuations within longer-term structural trends), and analytical resilience (a disposition to see ambiguous market conditions as a problem to be solved through systematic information gathering rather than as something to be avoided). These orientations of cognition matched well with the cognitive response scale’s flexibility and information integration sub-dimensions and provided a qualitative explanation of the quantitative patterns observed in Model 1.

#### 3.5.4 Theme 4: behavioral adaptation and strategic action

The fourth theme focuses on the behavioral aspects of participants’ reactions to green economic volatility, including financial and non-financial adaptations. The two main behavioral orientations identified were based on sub-theme analysis. Participants reported green investment pivoting, actively reallocating or diversifying their investment portfolios in response to new green market signals, and this was most observed among participants with high English proficiency and IT skills. Career and policy advocacy encompassed a broader set of adaptive behaviors, including making career pivots into sustainable finance and environmental governance, engaging in policy consultation processes, and creating digital content to communicate green economic literacy through professional networks. High dual competency participants tended to describe a coherent behavioral strategy, from personal investment toward collective institutional action, whereas low dual competency participants tend to describe reactive or passive behavioral orientations.

#### 3.5.5 Overarching theme: compounding effect of dual competency on sustainable economic engagement

The overall theme is present across all four main themes and captures the main finding of the qualitative analysis: the interplay of IT competency and English proficiency resulted in a qualitatively different level of engagement with green economic volatility, achieved by neither competency alone. Those who achieved high scores on both dimensions found the integration of digital tools and knowledge access via language to be smooth and connected, enabling them to form coherent, globally informed, and strategically adaptive responses to market uncertainties. This is qualitative evidence that complements and enriches the statistically significant compounding interaction effect found in both regression models and the SEM, and enhances the interpretability of the compounding quantitative interaction effect (Christodoulou-Volos, 2026). The thematic map in Figure 5 illustrates the interrelationships of themes and their relationship to the overarching finding.

#### 3.5.6 Integration of quantitative and qualitative findings

Consistent with the sequential explanatory design, the qualitative themes were integrated with the quantitative findings to clarify why the statistical associations emerged (Mattiello, 2024). Theme 1 (Digital Navigation of Green Market Information) explains the strong association between IT competency and cognitive response by showing how participants with stronger digital skills searched, filtered, and compared green-market information more effectively. Theme 2 (English as a Gateway to Global Green Knowledge) explains why English proficiency was the strongest predictor of behavioral response: participants with stronger English skills reported direct access to international policy reports, green-bond documents, and sustainability disclosures that informed adaptive action. Themes 3 and 4 correspond to the cognitive-flexibility and behavioral-adaptation sub-dimensions, respectively, and show how competence translated into confidence, information-seeking, investment adjustment, career adaptation, and policy engagement. Finally, the overarching theme of compounding dual competency contextualizes the statistically significant IT × English interaction observed in both the regression and SEM models. Thus, the qualitative phase does not simply repeat the quantitative results; it provides mechanism-level explanation and strengthens the inferential coherence of the mixed-methods design.

### 3.6 Summary of hypothesis testing outcomes

H1 was supported: IT competency was the strongest predictor of cognitive response in both the regression model (*β* = 0.41, *p* < 0.001) and SEM (*β* = 0.41, *p* < 0.001). Qualitative evidence explained this association through digital information navigation, source evaluation, and analytical confidence. H2 was supported: English proficiency was the strongest predictor of behavioral response (*β* = 0.39, *p* < 0.001), and interview data showed that English proficiency enabled access to international green finance, climate-policy, and sustainability-disclosure information. H3 was also supported: the IT × English interaction was significant in the cognitive response model (*β* = 0.22, *p* < 0.001) and the behavioral response model (*β* = 0.26, *p* < 0.001), indicating that digital and linguistic competencies were jointly associated with stronger adaptive responses.

H4 was evaluated as a control-variable hypothesis. Educational level explained a small but significant amount of additional variance in cognitive response (*β* = 0.08, *p* = 0.033) and behavioral response (*β* = 0.09, *p* = 0.018), whereas age and gender did not show significant independent associations after adjustment for the main predictors. These findings indicate that demographic characteristics made a modest contribution to the models, especially educational level, but the primary explanatory patterns were carried by IT competency, English proficiency, GEV perception, and the IT × English interaction. H5 was partially supported: the indirect pathways through GEV perception were significant for both outcomes (indirect *β* = 0.09 and 0.07), while direct paths remained significant, indicating partial mediation.

### 3.7 Integrated discussion

The findings advance the manuscript’s interdisciplinary argument by showing that IT competency and English proficiency function as complementary forms of human capital in the digital green economy. IT competency was most closely associated with cognitive response because volatile green-market information is increasingly encountered through digital dashboards, sustainability databases, visualization tools, and algorithmically mediated information streams. English proficiency, by contrast, was most closely associated with behavioral response because many authoritative green-economy resources are produced in English, including international policy reports, sustainability disclosures, and green-finance documentation.

The mixed-methods integration strengthens the interpretation of these associations. Quantitative models identified the magnitude and direction of the relationships, whereas interviews explained the processes through which these relationships operated. Participants with stronger IT skills described systematic search, comparison, and verification practices, supporting the cognitive-response pathway. Participants with stronger English skills described broader access to global knowledge sources and more confident translation of knowledge into action, supporting the behavioral-response pathway. Participants with high scores in both competencies described the most coherent adaptive strategies, which explains the significant IT × English interaction observed in both outcome models.

The results also clarify the role of demographic characteristics. Age, gender, and educational level were included as covariates to adjust for background heterogeneity, and the findings showed that educational level had a modest independent association with both outcomes. However, the central theoretical contribution lies in the competency-based pathways rather than demographic differences. This alignment between hypothesis formulation and statistical modeling improves internal consistency and avoids overextending the empirical claims beyond the tested model (Fetters et al., 2013).

In practical terms, the study suggests that future-ready education for the green economy should not treat digital literacy, English-language education, and sustainability knowledge as separate curricular domains. Instead, programs should integrate data literacy, English-mediated access to global knowledge, multimedia learning, and green-economy problem solving. This position is consistent with research on technology-mediated language learning, multimedia cognition, and future-oriented digital education (Mayer, 2005).

## 4 Conclusion

This study examined how IT competency and English-language education are associated with cognitive and behavioral responses to green economic volatility among 420 survey participants and 24 interview participants. The findings indicate that IT competency is more closely associated with cognitive outcomes, including risk appraisal, information integration, and cognitive flexibility, whereas English proficiency is more closely associated with behavioral adaptation through access to global green-economy knowledge. The significant IT × English interaction suggests that the two competencies operate synergistically rather than independently. The qualitative findings strengthened this interpretation by showing how participants used digital tools and English-language knowledge sources to move from information access to strategic action. By aligning the hypotheses, statistical model, and qualitative interpretation, the study contributes to human capital theory, digital transformation research, and English-language education for sustainable economic participation. Because the quantitative design was cross-sectional, the findings should be interpreted as associational rather than causal. Future longitudinal and cross-cultural studies should examine how integrated digital-English education shapes adaptive capacity during green economic transitions.

## Data Availability

The raw data supporting the conclusions of this article will be made available by the authors, without undue reservation.
